# Soybean green manure intercropping improves citrus quality by improving soil quality and altering microbial communities

**DOI:** 10.3389/fpls.2025.1560550

**Published:** 2025-08-28

**Authors:** Sufeng Deng, Binbin Huang, Bin Zeng, Sheng Cao, Biya Gong, Wei Liao, Wen Zhang, Sainan Luo, Shuizhi Yang

**Affiliations:** ^1^ Hunan Horticultural Research Institute, Yuelushan Laboratory, Changsha, China; ^2^ Hunan Provincial Engineering and Technology Research Center for Agricultural Microbiology Application, Hunan Institute of Microbiology, Yuelushan Laboratory, Changsha, China

**Keywords:** soybean green manure (SGM) intercropping, citrus quality, soil quality, soil microbial communities, community diversity, keystone microbial taxa

## Abstract

**Introduction:**

Intercropping leguminous green manure in orchards represents a widely adopted agroecological practice that concurrently influences soil physicochemical properties, microbial communities, and crop performance. However, the temporal mechanisms by which different durations of soybean green manure (SGM) intercropping regulate soil-plant-microbe interactions remain insufficiently understood. This study elucidates the impact of SGM intercropping duration on ecosystem functionality in citrus orchards.

**Methods:**

A multi-year field experiment compared SGM intercropping durations (0-, 1-, and 2-year treatments). We assessed citrus fruit quality parameters (total soluble solids, TSS; sugar-acid ratio, TSS/TA) and soil properties (pH, available nitrogen, total nitrogen, available phosphorus, available potassium, and organic matter). Microbial community structure was analyzed via high-throughput sequencing. Spearman correlation analysis (|*ρ*| ≥ 0.8, *p* < 0.05) delineated networks among intercropping duration, soil parameters, keystone microbial taxa (e.g., Proteobacteria, Acidobacteriota, Ascomycota), and fruit quality indicators.

**Results:**

The two-year intercropping treatment (T2) significantly enhanced fruit quality: TSS increased by 11.66% and the sugar-acid ratio (TSS/TA) by 41.95% (*p* < 0.05). Soil properties improved markedly: pH rose by 0.42 units, while AN, TN, AP, AK, and OM increased by 41.80%, 9.15%, 16.78%, 100.50%, and 79.53%, respectively (*p* < 0.05). Microbial communities underwent structural reorganization, exhibiting increased α-diversity, enhanced network complexity, and selective enrichment of beneficial taxa including Actinobacteria, Mortierellales, and Ascomycota. Correlation networks revealed significant associations among intercropping duration, soil parameters, keystone microbes, and fruit quality.

**Discussion:**

This study demonstrates that SGM intercropping enhances fruit quality through dual mechanisms: (1) amelioration of soil properties (pH elevation and improved nutrient availability), and (2) functional restructuring of microbial communities. Notably, specific taxa such as Actinobacteria play pivotal roles in nutrient cycling. Our findings provide empirical evidence for microbiome-mediated optimization of soil functionality, offering a sustainable rehabilitation strategy for degraded orchards and reinforcing the scientific value of ecological intensification in perennial cropping systems.

## Highlights

- Intercropping soybean green manure (SGM) enhanced the soil pH, organic matter content, and available nitrogen, available phosphorus, and available potassium concentrations.
- Intercropping SGM altered the soil microbial community composition and diversity, reconstructed co-occurrence networks, and enriched beneficial microbial taxa including *Actinobacteria*, *Proteobacteria*, *Ascomycota*, and *Mortierellales*.
- Intercropping SGM improved the citrus fruit quality, significantly increasing total soluble solid content (TSS) and TSS/titratable acidity ratio (TSS/TA).
- Citrus fruit quality exhibited strong correlations with intercropping duration (years), soil physicoproperties, and soil microbial community characteristics (diversity and specific microbial taxa).
- Intercropping SGM in orchards critically enhanced the soil quality and crop productivity, advancing sustainable agricultural practices.

## 1 Introduction

Citrus represents a significant economic fruit crop extensively cultivated in China, with documented planting areas and yields reaching 2.67 million hectares and over 600 million tonnes, respectively, in 2023 (Shen et al., 2024; National Bureau of Statistics of China, 2025). As a principal cultivar, navel orange (*C. sinensis* Osbeck) dominates production across key provinces including Jiangxi, Sichuan, Hubei, and Hunan (Yao et al., 2022; National Bureau of Statistics of China, 2025). Critical determinants of fresh fruit quality and commercial value encompass total soluble solids content (TSS), titratable acidity (TA), and sugar–acid ratio (TSS/TA) (Yu et al., 2015; Wu et al., 2021; Guo et al., 2023). The sensory quality and market value of navel oranges are largely determined by their TSS, TA, and TSS/TA, which collectively contribute to a balanced sweet–tart flavor profile. However, the accumulation of soluble sugars and total soluble solids (TSS) and the conversion of titratable acidity (TA) in fresh fruits are influenced by numerous factors, including the genetic characteristics of fruit trees, environmental variables (climate and soil conditions), and agricultural management practices (intercropping, crop rotation, no-till farming, and fertilization) as well as the timing of harvest (Li and Wan, 2006; Li C. et al., 2023; Yu et al., 2024). Intercropping has emerged as an efficacious approach for fostering the sustainable development of orchards and woodlands. This practice not only aids in minimizing the reliance on chemical fertilizers but also enhances crop yield and quality while simultaneously preserving the soil ecosystem (Li et al., 2020; Dong et al., 2021). The soil environment holds a pivotal position in determining the quality and productivity of orchards and woodlands, encompassing both the physical and chemical characteristics of the soil and the functional dynamics of the soil microbial community (Dong et al., 2021; Fu et al., 2023; Li C. et al., 2023).

Intercropping, a cornerstone of traditional Chinese agriculture with a history spanning over 2,000 years, has garnered significant attention due to its myriad benefits. These include mitigating crop competition for land, enhancing land utilization, stabilizing and augmenting yields per unit area, diminishing pest and disease issues, reducing agrochemical reliance, and fostering biodiversity (Li C. et al., 2023; Duan et al., 2024; Qin et al., 2024). Recently, the advancement and dissemination of modern orchard cultivation practices centered on wide-row, dense planting, mechanization, and intelligent, labor-saving technologies for citrus and other fruits in China have created conducive environments for orchard and woodland intercropping (Qin et al., 2024). Extensive literature attests to the prevalent practice of intercropping leguminous green manure in soil ecosystems, including farmland, orchards, and woodlands (Wang T. et al., 2022; Duan et al., 2024; Qin et al., 2024). Soybean and other leguminous crops, noted for their nitrogen-fixing capabilities, ease of cultivation, and management, are frequently employed as green manure or intercrops for balanced fertilization and soil amelioration (Jing et al., 2022; Yu et al., 2025). Research indicates that intercropping leguminous green manure in tea plantation woodlands alters the soil bacterial community, influencing amino acid metabolism and flavonoid biosynthesis, ultimately enhancing tea quality (Wang T. et al., 2022; Duan et al., 2024; Yu et al., 2025). This practice also modulated soil physicochemical properties, boosted soil nutrient metabolism, and improved soil quality. Furthermore, soil microorganisms serve as vital bio-indicators reflecting soil health, plant growth, and development. They participated in nutrient cycling and impacted crop resistance to disease and stress, which was pivotal for fostering healthy crop growth, sustainable agricultural development, and ecosystem stability (Jing et al., 2022; Li C. et al., 2023). The structure and function of soil microbial communities were comprehensively influenced by soil properties, the soil environment, and agronomic practices (Jing et al., 2022; Wang T. et al., 2022; Duan et al., 2024; Yu et al., 2025). Studies highlighted the crucial role of soil fungal communities in regulating ecosystem services related to soil fertility, while bacterial communities were intimately linked to plant growth and soil properties (Read and Perez, 2003; Kyaschenko et al., 2017).

TCurrently, despite the fact that the utilization of chemical fertilizers can sustain the productivity of citrus navel orange orchards, the prolonged and excessive application of these fertilizers in orchards with acidified soils adversely affects the physicochemical properties of orchard soils, soil pH, and soil microbial communities (Lin et al., 2019; Wang Z. et al., 2022; Raiesi et al., 2024). Such practices are unsustainable for long-term development. To mitigate these issues, studies on intercropping leguminous green manures in orchards or woodlands have demonstrated that this practice can enrich soil nutrients, modulate soil properties and microbial communities, and enhance crop yield and quality (Wang T. et al., 2022; Wang Z. et al., 2022; Duan et al., 2024; Raiesi et al., 2024; Yu et al., 2025). Consequently, this study examined the effects of intercropping soybean green manure (SGM) in citrus orchards on orchard ecological functions (fruit yield and quality) and orchard soil ecosystems (soil properties, soil microbial communities) over different durations (0, 1, and 2 years). Additionally, the study evaluated the correlations between intercropping duration, citrus yield and quality, soil properties, and soil microbial communities. Ultimately, it is anticipated that the findings of this research will furnish theoretical backing for the establishment of a green manure model aimed at intercropping soybean in citrus orchards located in acid soil regions.

## 2 Materials and methods

### 2.1 Site description and experimental design

TThe citrus orchard experiment was situated in Houjiawan Village, Yongding District, Zhangjiajie City, Hunan Province, China (110°42′ E, 29°11′ N), within a subtropical mountain monsoon humid climate zone characterized by an average annual sunshine duration of 1,440 h, a mean temperature of 17°C, annual precipitation of 1,400 mm, and a frost-free period of 216–269 days. The gently sloping and flat orchard covered approximately 2 ha of uniform acidic brown soil, with 6-year-old Newhall navel orange trees (2022) spaced at 3 m × 4 m (row spacing). The experimental groups included T0 (control, no SGM intercropping or tillage in 2021–2022), T1 (no treatment in 2021, soybean intercropping and tillage in 2022), and T2 (two consecutive years of soybean intercropping and tillage in 2021 and 2022), each comprising five replicate plots of 20 trees (four rows × five trees/row) separated by two buffer tree rows to minimize cross-plot interference. Quarterly weed mowing was implemented, while the citrus trees received annual fertilization in April with 10 kg of organic fertilizer (2.5% N, 2.0% P_2_O_5_, 2.8% K_2_O) and 2 kg of compound fertilizer (15-15-15, N–P_2_O_5_–K_2_O) per tree, applied to 30-cm-deep furrows aligned with drip lines; for the soybean (*Glycine max L*. cv. Xiangchun 24) intercropping system, six rows were planted in 1.5-m-wide strips between citrus rows in March without supplemental fertilization, with residues mechanically processed into 3–5-cm segments and incorporated into topsoil (0–20-cm depth) via rotary tiller after fresh pod harvest in late July.

### 2.2 Soil sampling and DNA sequencing

On November 12, 2022 (fruit harvest date), soil samples were randomly collected from five points in each of the five experimental fields per treatment (totaling 15 samples), following modified protocols from Zhou et al. (2021) and Wu et al. (2024). The sampling avoided treatment edges and fertilizer application zones, specifically targeting the 5–15-cm depth within the 5–20-cm soil layer where citrus roots and soybean residues interact. All samples were immediately transported to the laboratory, where each was divided: one portion preserved for DNA extraction and sequencing, while the other was air-dried for physicochemical analysis.

TMicrobial DNA was extracted from soil samples using the E.Z.N.A.^®^ Soil DNA Kit (Omega Bio-tek, Norcross, GA, USA) following the manufacturer’s protocols. PCR amplification was conducted using the Takara ExTaq PCR kit (Takara Shuzo, Osaka, Japan) with specific primers: 338F (5′-ACTCCTACGGGAGGCAGCAG-3′) and 806R (5′-GGACTACHVGGGTWTCTAAT-3′) for bacterial 16S rRNA V3–V4 regions and ITS1F (5′-CTTGGTCATTTAGAGGAAGTAA-3′) and ITS2R (5′-GCTGCGTTCTTCATCGATGC-3′) for fungal ITS regions. The thermal cycling protocol consisted of initial denaturation at 95°C for 2 min, followed by 25 cycles of 95°C for 30 s, 55°C for 30 s, and 72°C for 30 s, with a final extension at 72°C for 10 min. PCR products were verified by 2% agarose gel electrophoresis, purified using a gel recovery kit, and sequenced on an Illumina MiSeq platform (Novogene, Beijing). The raw sequencing data were deposited in the NCBI Sequence Read Archive (accession: PRJNA957046).

### 2.3 Fruit yield and quality evaluation

At the stage of edible maturity (November 12), fruit sampling was performed using a stratified protocol: five fruits were randomly collected from different crown aspects of each tree, with three trees randomly selected per plot, yielding 15 fruits per plot across four replicate plots per treatment group, thus totaling 60 fruits per treatment. The yield and quality parameters of citrus were determined according to GB/T 12947-2008 (Fresh Citrus Fruits: National Standards Information Public Service Platform) (China National Standardization Administration Committee, 2008) as briefly described below: single fruit weight (SFW) was directly measured using an electronic balance; yield per tree (YPT) was calculated by multiplying the mean SFW by the total fruit count per tree (field-recorded); and for quality assessment, 15 fruits per plot were pooled, peeled, and juiced with a mechanical extractor. Total soluble solids (TSS), expressed as a percentage (%) and representing water-soluble components (sugars, acids, vitamins, minerals), were measured using a handheld refractometer (PAL-BXIACID1, 0%–90%; Atago Co., Japan); titratable acidity (TA), quantifying total organic acids in the juice, was determined via the refractometer’s acid titration mode; and the TSS/TA ratio was derived from the relative proportion of soluble solids to organic acids.

### 2.4 Analysis of soil physicochemical properties

The determination of soil chemical properties followed the procedures described in Soil Agrochemical Analysis edited by Bao SD (Bao, 2000), detailed as follows: Soil pH was measured using a pH meter with a soil-to-deionized water ratio of 1:2.5 (w/v). Organic matter content was determined via the potassium dichromate–sulfuric acid oxidation method. Total nitrogen was quantified using the Kjeldahl method, total potassium by flame photometry, and total phosphorus by molybdenum blue colorimetry. For available nutrients, alkali-hydrolyzable nitrogen was measured using the alkaline hydrolysis diffusion method, available potassium via neutral ammonium acetate extraction–flame photometry, and available phosphorus through sodium bicarbonate extraction–molybdenum antimony resistance colorimetry.

### 2.5 Soil microbial community analysis

The bioinformatics analysis of high-throughput sequencing results was performed following the methods described by Zhou et al. (2021) and Wu et al. (2024). Briefly, raw data were cleaned to remove assembly artifacts and then compared against the SILVA Database v138.1 and UNITE v7.2 Database for species annotation of bacteria and fungi, respectively. These results were further utilized for downstream analysis. Linear discriminant analysis effect size (LEfSe) with a linear discriminant analysis (LDA) threshold of 4.0 was employed to identify genus-specific microbes in each group. Phylogenetic tree diagrams were used to visualize differences in microbial communities from the phylum to species level. All of these data analyses were performed online on the NovoCloud Platform (https://magic.novogene.com) for both analysis and visualization.

### 2.6 Co-occurrence network analysis

The co-occurrence network analysis based on Spearman’s correlation matrix was conducted using the “Hmisc” package (version 5.2.3) (Harrell, 2019) in R software. To ensure accuracy, amplicon sequence variants (ASVs) present in at least three subsamples within each group and with relative abundances >0.1% were included in the analysis. The results were filtered using thresholds of |*ρ*| >0.8 and *p <*0.05. Network visualization was performed in Gephi (V0.9.2) (Bastian et al., 2009) with the Fruchterman Reingold layout and network topological parameters, which included nodes, edges, positive edges, negative edges, average degree, average clustering coefficient, and average path length, were calculated in Gephi.

### 2.7 Statistical analysis

Data processing was conducted using Excel 2020 software, with values in tables presented as means ± standard error. To analyze significant differences in soil properties and alpha diversity indices among all treatment groups, one-way analysis of variance (ANOVA) combined with Duncan’s multiple-range test was employed. Statistical significance was determined at *p*-values <0.05. Additionally, analysis of similarity (ANOSIM) was performed to ascertain whether intergroup differences were significantly greater than intragroup variations. Venn diagrams were constructed using the R package “ggvenn” (version 0.1.9); note that a previous mention of “vegan” for this purpose was incorrect. Bacterial diversity analysis was facilitated by the R package “tidyverse” (version 2.0.0). Correlation analysis was carried out using the R package “Hmisc” (version 5.2.3), with subsequent network visualization performed in Cytoscape (version 3.5.0). Other data analyses and visualizations, including histograms of community composition, were executed on the Novogene Cloud Platform (https://magic.novogene.com).

## 3 Results

### 3.1 Citrus yield and quality

The effects of intercropping SGM with different years on citrus yield and quality parameters are presented in **Table 1**. As the years of intercropping SGM increase (0–2 years), the citrus trees showed progressive increases in yield per tree (YPT), single fruit weight (SFW), total soluble solids (TSS), and TSS/titratable acidity (TSS/TA) ratio. Conversely, titratable acidity (TA) exhibited a gradual decline. Crucially, significant improvements (*p* < 0.05) in fruit quality were observed exclusively for TSS and TSS/TA following 2 years of SGM intercropping (T2) that fruit TSS increased by 11.66% from 11.97% (T0) to 13.37% (T2) and TSS/TA rose markedly by 41.95% from 15.59 (T0) to 22.12 (T2). Although the YPT, SFW, and TA showed favorable directional changes (increases for YPT/SFW, decrease for TA), these alterations failed to reach statistical significance. So, the results indicate that intercropping SGM enhanced citrus fruit quality, with the most pronounced improvements in TSS and TSS/TA occurring specifically after 2 years of implementation (T2).

**Table 1:** Effect of SGM intercropping with different years on citrus yield and quality.

Treatments	Yield		Quality		
	Yield per tree (YPT), kg	Single fruit weight (SFW), g	Total soluble solid (TSS), %	Total acid (TA)	Solid–acid ratio (TSS/TA)
T0	27.79 ± 1.69 a	158.91 ± 7.37 a	11.97 ± 0.43 b	0.73 ± 0.08 a	15.59 ± 1.16 b
T1	28.90 ± 1.20 a	164.58 ± 7.54 a	12.61 ± 0.81 ab	0.70 ± 0.02 a	18.23 ± 1.26 b
T2	29.98 ± 2.08 a	167.79 ± 7.64 a	13.37 ± 0.56 a	0.66 ± 0.05 a	22.12 ± 3.79 a

The data shown are mean ± standard error. The lowercase letters denote significant differences between treatments as determined by Duncan’s tests (*p* < 0.05).

### 3.2 Soil physicochemical properties

As shown in **Table 2**, intercropping SGM in citrus orchards differentially impacted soil physicochemical properties across treatment durations (0–2 years). The orchard soils exhibited acidity, with pH values ranging from 5.11 to 5.53. With increasing intercropping duration, significant enhancements (*p* < 0.05) were observed in soil pH, available nitrogen (AN), total nitrogen (TN), available phosphorus (AP), available potassium (AK), and organic matter (OM) content. However, no significant differences occurred in total phosphorus (TP) or total potassium (TK). Notably, at 1 year of SGM intercropping treatment (T1), significant increases (*p* < 0.05) were observed in soil pH (+0.26 units), available nitrogen (AN, +17.42%), available phosphorus (AP, +13.86%), available potassium (AK, +62.75%), and organic matter (OM, +28.52%). After 2 years of treatment (T2), further significant enhancements occurred in soil pH (+0.16 units), AN (+20.77%), AK (+23.20%), and OM (+39.69%). Cumulatively, the T2 treatment significantly improved the soil pH (+0.42 units), AN (+41.80%), total nitrogen (N, +9.15%), AP (+16.78%), AK (+100.50%), and OM (+79.53%) compared with the control (T0) (*p* < 0.05). Thus, SGM intercropping improved the soil physicochemical properties in citrus orchards, with particularly significant enhancements in soil pH and fertility indicators (AN, AK, AP, TN, OM) following 2 years of implementation.

**Table 2:** Effect of SGM intercropping with different years of treatment on soil properties.

Treatments	pH	Available nitrogen (AN)	Nitrogen (N)	Available phosphorus (AP)	Phosphorus (P)	Available potassium (AK)	Potassium (K)	Organic matter (OM)
		mg/kg	g/kg	mg/kg	g/kg	mg/kg	g/kg	g/kg
T0	5.11 ± 0.01 c	97.6 ± 8.85 c	1.53 ± 0.07 b	28.78 ± 0.26 b	2.11 ± 0.19 a	80.0 ± 14.76 c	13.20 ± 0.8 a	20.86 ± 2.7 c
T1	5.37 ± 0.03 b	114.6 ± 8.96 b	1.60 ± 0.05 ab	32.77 ± 1.51 a	2.10 ± 0.09 a	130.2 ± 7.33 b	12.92 ± 0.53 a	26.81 ± 4.05 b
T2	5.53 ± 0.02 a	138.4 ± 9.69 a	1.67 ± 0.09 a	33.61 ± 0.76 a	2.14 ± 0.07 a	160.4 ± 11.04 a	13.86 ± 0.76 a	37.45 ± 3.28 a

The data shown are mean ± standard error. The lowercase letters denote significant differences between treatments as determined by Duncan’s tests (*p* < 0.05).

### 3.3 Soil microbial composition and diversity

#### 3.3.1 Soil microbial composition

A total of 1,168,771 and 1,068,647 high-quality bacterial and fungal sequences were obtained after sequencing and quality control, respectively. There were 59,960–72,770 and 55,850–83,106 valid reads obtained in bacterial and fungal ASVs, respectively. The corresponding rarefaction curves tended to nearly saturate at the selected sequencing depth (47,940 and 48,772 bacterial and fungal valid reads ASVs, respectively; **Supplementary Figures S1**, **S2**; **Supplementary Tables S1**, **S2**).

**Figure 1:**
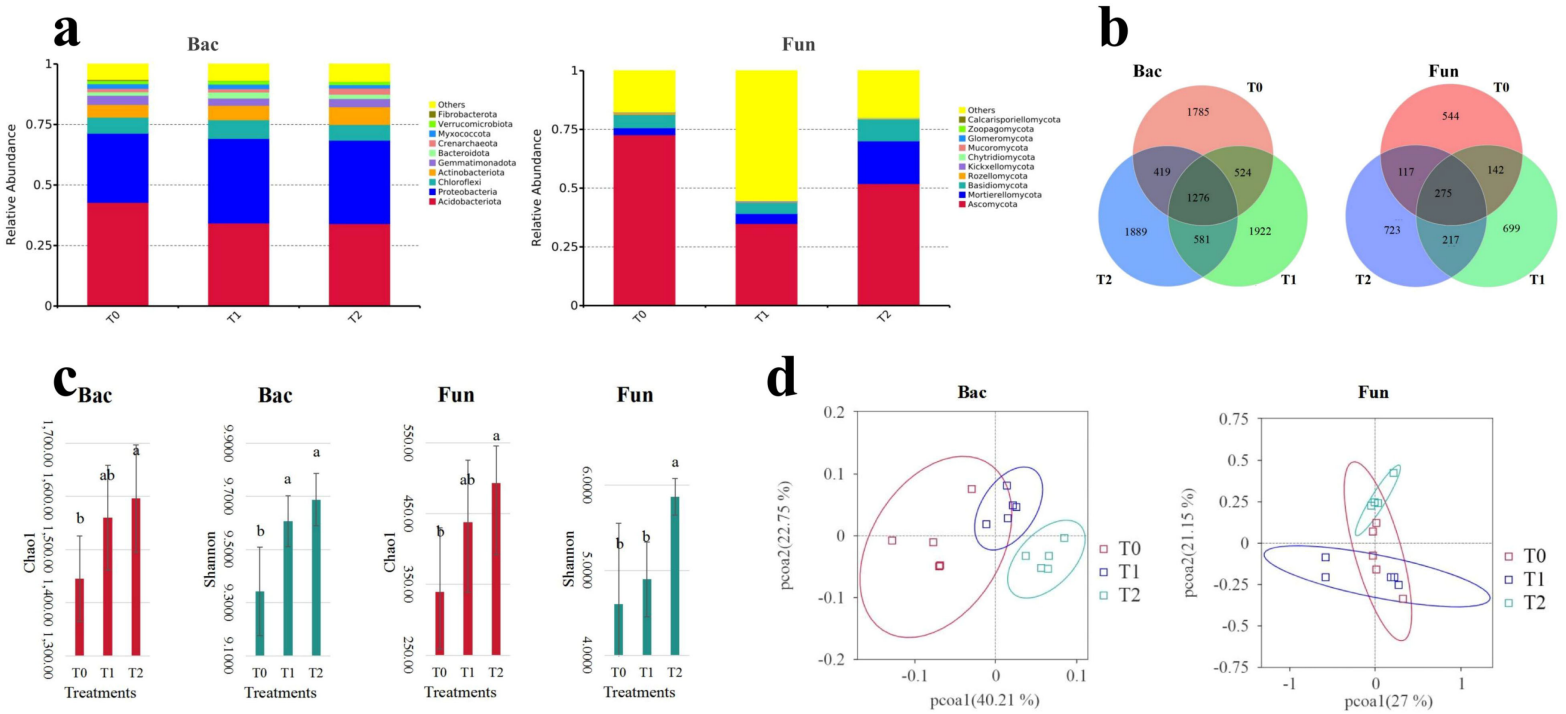
Soil bacteria (Bac) and fungal (Fun) communities of the top 10 phyla: relative abundance **(a)**, Veen analysis **(b)**, principal coordinate analysis **(c)**, and alpha-diversity **(d)**.

Among three different treatments, the dominant phyla (abundance in top 10) of soil bacterial communities (**Figure 1a**; **Supplementary Table S3**) were *Acidobacteriota* (34.03%–42.81%), *Proteobacteria* (28.54%–34.90%), *Chloroflexi* (6.52%–7.75%), *Actinobacteriota* (5.22%–7.29%), *Gemmatimonadota* (2.98%–3.80%), *Bacteroidota* (1.45%–2.48%), *Crenarchaeota* (1.41%–2.55%), *Myxococcota* (1.44%–1.92%), *Verrucomicrobiota* (1.30%–1.49%), and *WPS-2* (1.03%–1.41%). Soil fungal communities (**Figure 1a**; **Supplementary Table S4**) were dominated by *Ascomycota* (34.92%–72.71%), *Mortierellomycota* (2.95%–18.16%), *Basidiomycota* (4.75%–9.30%), *Rozellomycota* (0.11%–0.61%), *Kickxellomycota* (0.04%–0.32%), *Chytridiomycota* (0.06%–0.32%), *Mucoromycota* (0.08%–0.09%), *Glomeromycota* (0.02%–0.11%), *Zoopagomycota* (0.00%–0.01%), and *Aphelidiomycota* (0.00%–0.01%). The Venn analysis of soil microbial communities (**Figure 1b**; **Supplementary Table S5**) revealed that intercropping SGM (T1, T2) increased the number of ASVs in both bacterial and fungal communities compared to the control (T0). However, after 2 years of SGM intercropping (T2), the ASV richness of soil bacterial and fungal communities declined relative to T1, and it was still higher than in T0. A principal coordinates analysis (PCoA) of soil microbial β-diversity based on weighted UniFrac distances revealed distinct separation between T0 (control) and T2 (2-year SGM intercropping) treatments (**Figure 1d**). However, partial overlap persisted between T1 (1-year SGM intercropping) and both T0 and T2 groups, indicating incomplete community differentiation. The cumulative explanatory power of the first two principal coordinates (PCo1 + PCo2) approached or exceeded 50% of total variation, accounting for 62.96% (bacteria: 40.21% + 22.75%) and 48.15% (fungi: 27.00% + 21.15%) in bacterial and fungal communities, respectively. All of these findings demonstrate that SGM intercropping restructures soil microbial composition, with significantly more pronounced shifts in both bacterial and fungal community architecture following a 2-year implementation.

#### 3.3.2 Soil microbial alpha diversity

Analyses of α-diversity indices (Chao1, observed species, Simpson, and Shannon) for soil bacterial and fungal communities revealed consistent increases under SGM intercropping treatments (T1, T2) compared to the monoculture control (T0) (**Figure 1c**; **Supplementary Table S6**). Notably, the T2 treatment (2-year intercropping) induced significant elevations (*p* < 0.05) in both Chao1 and Shannon indices for soil bacterial and fungal communities. In contrast, only the Simpson index of bacterial communities exhibited a significant enhancement (*p* < 0.05) following a 1-year intercropping (T1). Thus, intercropping SGM in citrus orchards enhanced the alpha diversity indices of soil bacterial and fungal communities, particularly after 2 years of implementation (T2) (*p* < 0.05).

### 3.4 Different taxon of soil bacterial and fungal communities

The unique ASV numbers of soil bacterial and fungal community are shown in **Figure 1b**. Compared to the no-intercropping treatment (T0), intercropping treatments (T1 and T2) increased the unique ASV numbers for both communities (**Figure 1b**; **Supplementary Table S5**). The number of unique ASVs increased as the intercropping years decreased, except for the fungal community, and this pattern was generally consistent with the overall changes in ASVs within the community.

**Figure 2:**
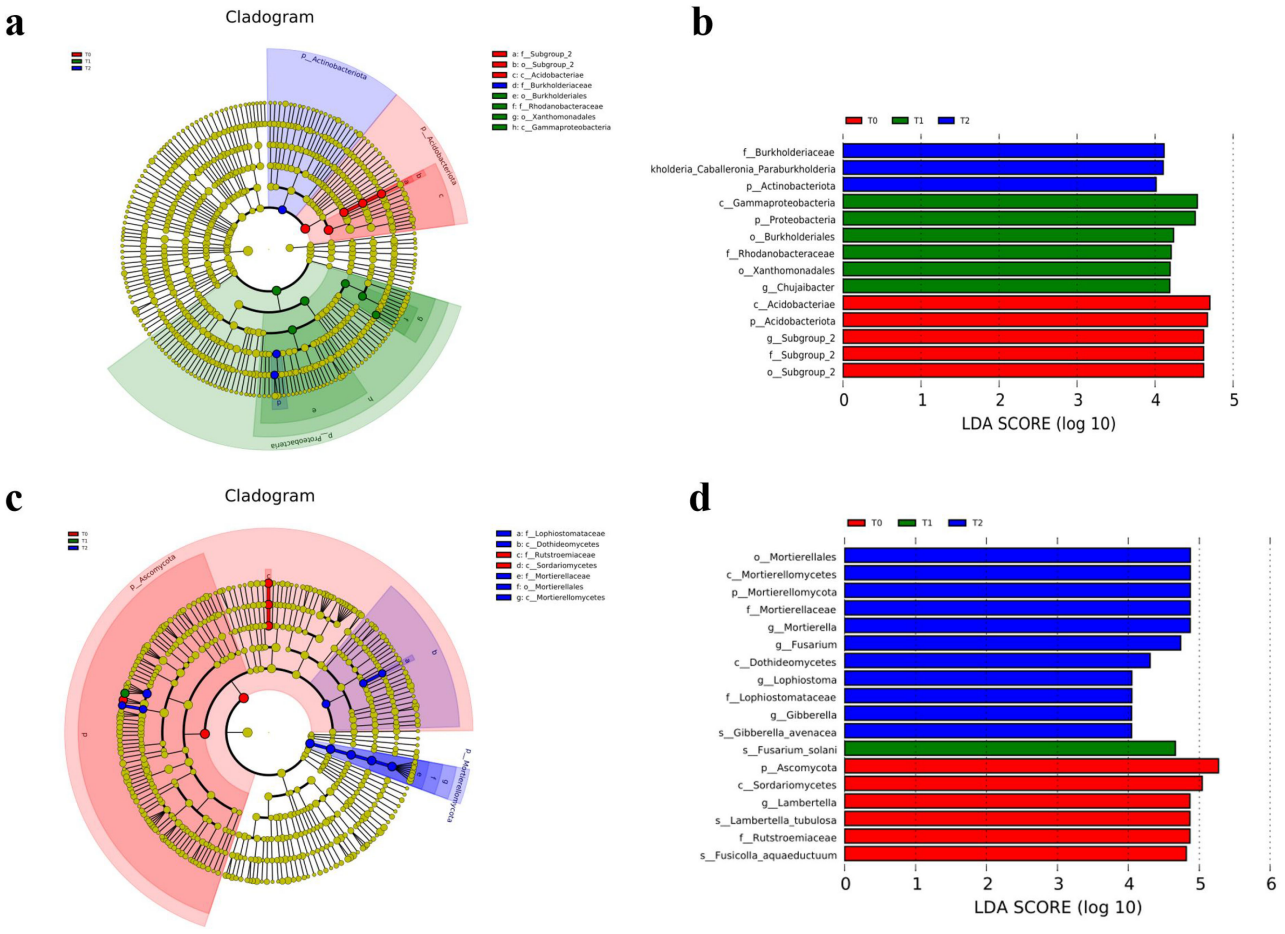
LEfSe and cladogram on different years of intercropping treatments of soil bacterial **(a, b)** and fungal **(c, d)** communities (LDA scores ≥4.0).

The cladogram analysis (**Figures 2a, c**) showed the result of linear discriminant analysis effect size (LEfSe, LDA scores ≥ 4.0) and indicated the taxonomic component of bacterial and fungal microbial communities. It showed a better visualization of shifts in the bacterial and fungal community in citrus orchard soil, whether T0, T1, or T2 treatment. For soil bacterial community (**Figure 2a**), *Acidobacteria* (from phylum to genus) and *Proteobacteria* (from phylum to genus) were significantly enriched in T0 and T1 treatments, respectively. However, *Actinobacteriota* (the phylum) and Burkholderiales (from family to genus) were significantly enriched in T2 treatment. For soil fungal communities (**Figure 2d**), *Ascomycota* (the phylum and its order *Sordariomycetes*) and *Rutstroemiaceae* (from family to species) were significantly enriched in T0 treatment. *Fusarium solani* (the species) was significantly enriched in T1 treatment. *Mortierellomycota* (from phylum to genus) and *Dothideomycetes* (the class and its family *Lophiostomataceae*) were significantly enriched in T2 treatment.

The LEfSe analysis revealed differential microbial taxa across treatments (**Figures 2b, d**). In the soil bacterial community, 14 discriminative taxa were identified (LDA score ≥ 4.0), with enrichment patterns distributed as follows: five in T0 (control), six in T1 (intercropping = 1 year), and three in T2 (intercropping = 2 years). The predominant bacterial phyla were *Acidobacteriae* in T0, *Proteobacteria* in T1, and *Actinobacteriota* in T2. For fungal communities, 18 discriminative taxa were detected (LDA score ≥ 4.0), exhibiting distinct treatment enrichment: six in T0, one in T1, and 11 in T2. The predominant fungal taxa were *Ascomycota* in T0, *Fusarium solani* (species taxon) in T1, and *Mortierellales* (order taxon) in T2.

### 3.5 Co-occurring network analysis

**Figure 3:**
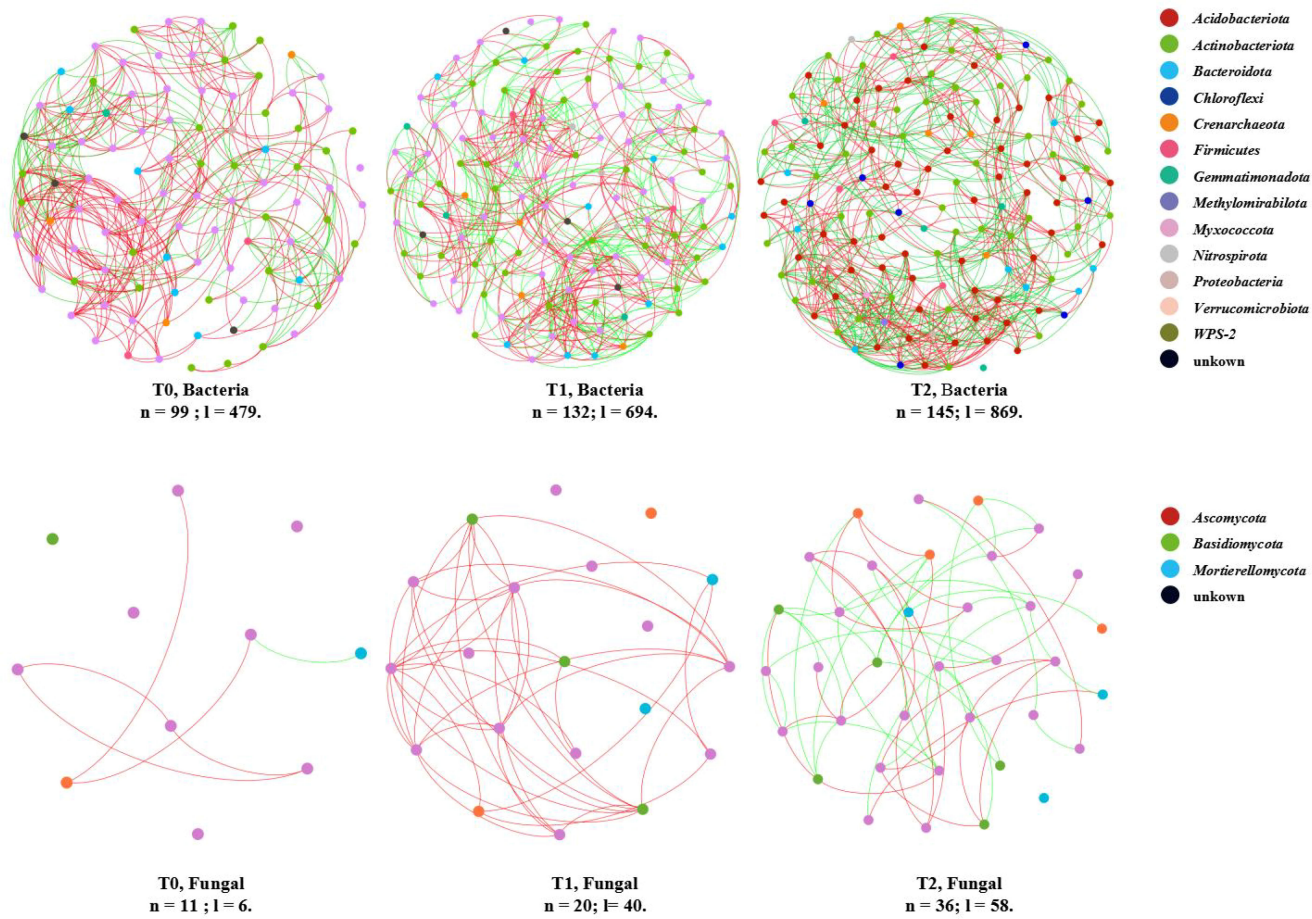
Co-occurring network of soil bacterial and fungal communities at ASV taxon. The nodes (n) are colored by phylum. The edges (l) show significant Spearman correlations (|*ρ*| > 0.8, *p* < 0.05), with red/green indicating positive/negative correlations.

The co-occurrence network was used to analyze the co-occurrence characteristics of intercropping SGM treatments on soil microbial community in citrus orchards. Soil bacterial and fungal communities’ ASVs with relative abundance ≥0.1% in three groups were constructed (**Figure 3**; **Supplementary Table S7**). The co-occurrence networks of soil bacterial and fungal communities exhibited increased node counts, link numbers, and structural complexity with extended intercropping duration (years) with SGM. Network connections shifted from predominantly positive links toward balanced positive–negative interactions (approaching 50%), indicating a transition from cooperative-dominant to cooperative-competitive dynamics, except for fungal communities in the T1 treatment. The average path length, inversely related to network compactness and stability (Li J. et al., 2023; Qiao et al., 2024), decreased progressively in bacterial networks (T0: 3.649; T1: 3.590; T2: 3.510), demonstrating enhanced cohesion and robustness over the intercropping chronosequence. Conversely, fungal networks displayed increased path lengths (T0: 1.444; T1: 1.725; T2: 3.651), reflecting gradual structural decentralization.

Elevated taxonomic diversity within these co-occurrence networks amplified complexity and critically supported soil microbial multifunctionality (Chen et al., 2022; Wang et al., 2023; Xiao et al., 2025). This complexity was evidenced by (i) increased node and link counts across bacterial and fungal networks (**Supplementary Table S7**; **Figure 3**) and (ii) declining proportional representation of dominant phyla alongside rising contributions from minor taxa. For bacterial networks (**Supplementary Figure S3**; **Supplementary Table S8**), nodes affiliated with *Acidobacteriota* decreased significantly (T0: 52.53% → T1: 44.70% → T2: 39.31%), whereas those assigned to *Proteobacteria* increased progressively (T0: 28.28% → T1: 34.09% → T2: 37.24%). Concurrently, the number of represented phyla expanded (T0: 9 → T1: 11 → T2: 11). Fungal networks exhibited parallel trends (**Supplementary Figure S4**; **Supplementary Table S9**).

Collectively, SGM intercropping in citrus orchards enhanced the structural complexity of soil microbial co-occurrence networks. Crucially, it increased species composition diversity within these networks, driving a shift from cooperation-dominated to balanced cooperative–competitive frameworks and reinforcing soil microbial community multifunctionality.

### 3.6 Correlations between soil properties and soil microorganisms

**Figure 4:**
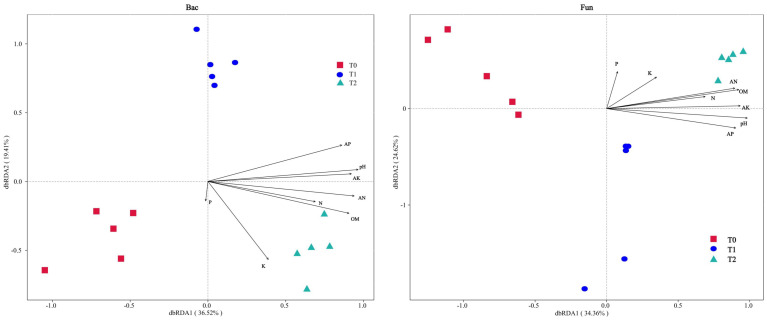
Distance-based redundancy analysis (dbRDA) for soil bacterial (Bac) and fungal (Fun) communities associated with soil properties at the ASV taxonomic level, respectively.

Distance-based redundancy analysis (db-RDA) was performed to assess the influence of soil properties on microbial communities (**Figure 4**). The first two axes collectively explained 55.93% (bacteria) and 58.98% (fungi) of community variation, indicating a robust model representation of environmental effects (>50% threshold). Bacterial and fungal communities exhibited directional succession along the dbRDA1 axis (left to right) with increasing SGM intercropping duration (0→1→2 years). Key drivers of community restructuring (*p* < 0.05, **Supplementary Table S10**) included soil pH, total nitrogen (N), available nitrogen (AN), available phosphorus (AP), available potassium (AK), and organic matter (OM). Notably, pH, AN, AK, and OM emerged as shared strong drivers for both communities (*p* < 0.01, r2 ≥ 0.8). In addition, acute angles between these factor vectors in ordination space implied synergistic interactions among dominant drivers.

**Figure 5:**
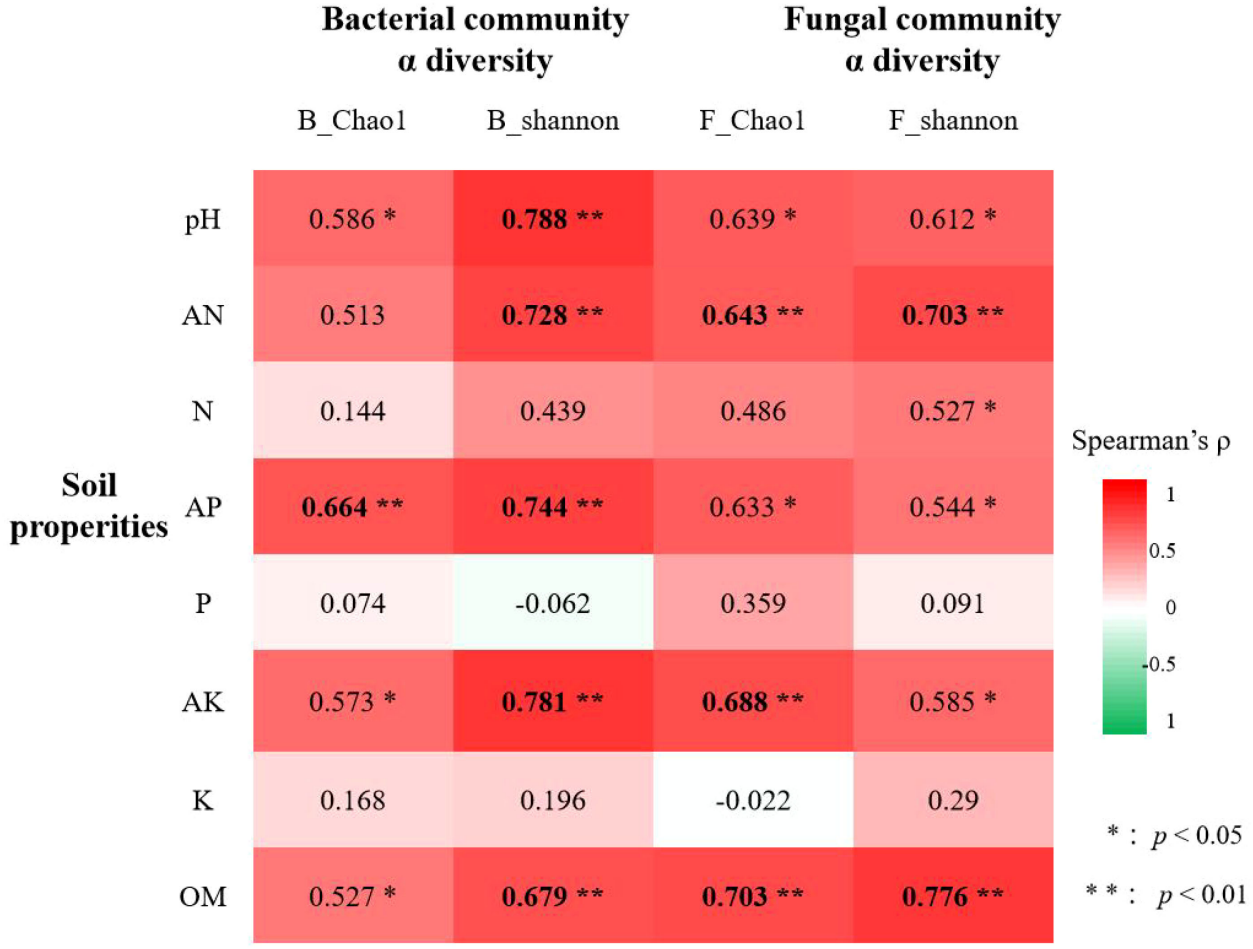
Correlations between soil properties and microbial α-diversity (Spearman’s |*ρ*| > 0.6 in bold).

Moreover, the correlation analysis between alpha diversity indices and the soil properties ae shown in **Figure 5**; **Supplementary Table S11**. For bacterial α-diversity, significant positive correlations were observed with soil pH, available nitrogen (AN), available phosphorus (AP), available potassium (AK), and organic matter (OM) (*p* < 0.05). The Shannon index exhibited particularly strong associations (*p* < 0.01) with soil pH (*ρ* = 0.788), AN (*ρ* = 0.728), AP (*ρ* = 0.744), and AK (*ρ* = 0.781). Similarly, fungal α-diversity showed significant positive correlations (*p* < 0.05) with soil pH, AN, AP, AK, and OM, with the Shannon index demonstrating robust linkages (*p* < 0.01) to AN (*ρ* = 0.703) and OM (*ρ* = 0.776). In contrast, total soil nitrogen (N), phosphorus (P), and potassium (K) displayed non-significant correlations (*p* > 0.05). Collectively, soil pH, available nutrients (AN, AP, AK), and OM emerged as key drivers shaping the α-diversity in both bacterial and fungal communities, particularly for the Shannon index. Notably, bacterial diversity displayed stronger correlations (higher coefficients, lower *p*-values) with soil pH, AN, AP, and AK than fungal diversity, whereas soil OM exerted a more pronounced influence (higher coefficient, lower *p*-value) on the fungal Shannon index.

**Figure 6:**
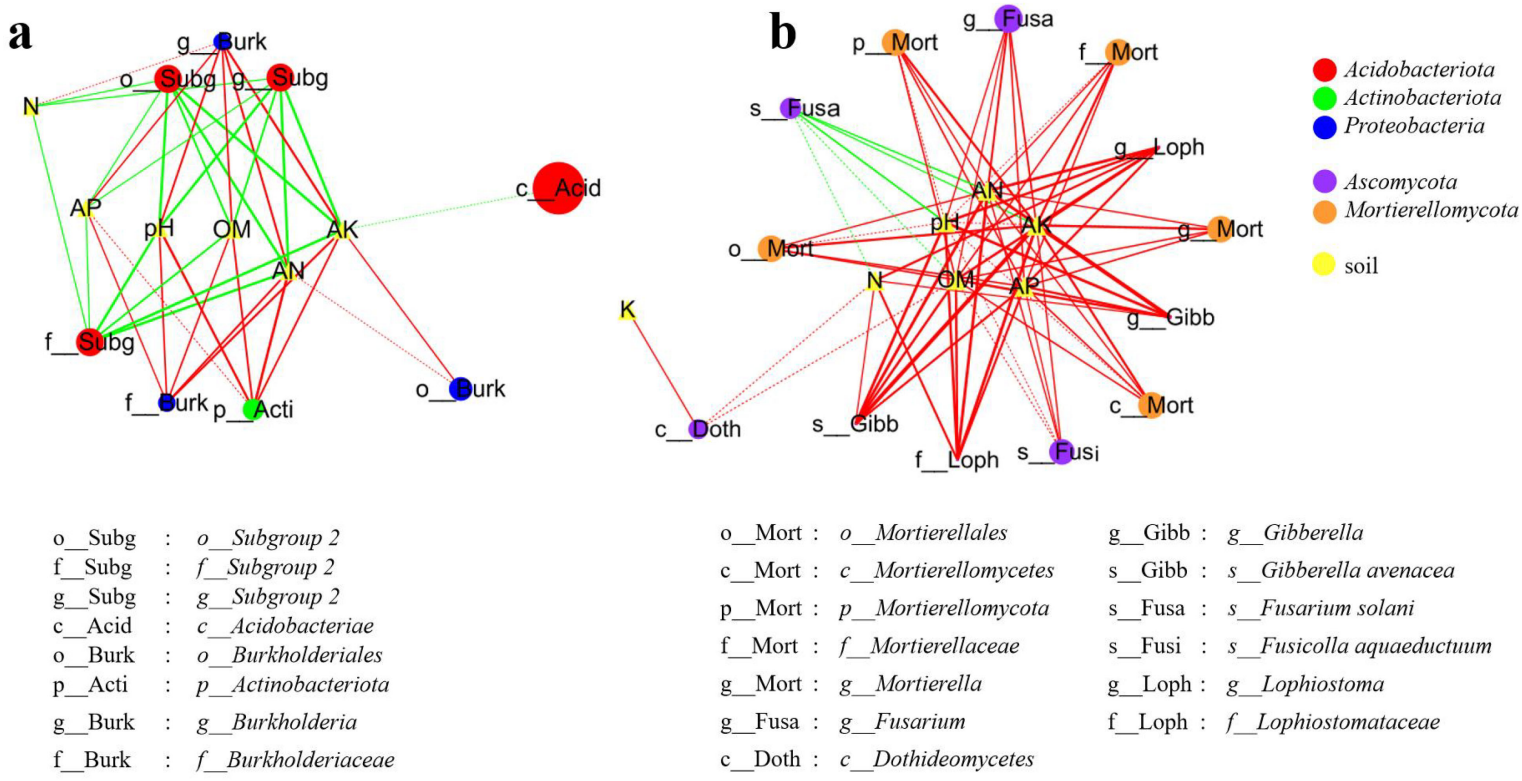
Correlations between soil properties and taxon-specific microbes (LEfSe, LDA ≥ 4.0; Spearman’s |*ρ*| ≥ 0.6 shown). The node size reflects microbial relative abundance. The solid line and dashed line represent *p* < 0.01 and *p* < 0.05, respectively; line with green (negative) and red (positive). The bold lines denote strong correlations (|*ρ*| ≥ 0.8).

Significant correlations (Spearman’s |*ρ*| ≥ 0.6, *p* < 0.05) between soil physicochemical properties and taxon-specific bacterial (**Figure 2b**) and fungal (**Figure 2d**) microbes are visualized in **Figure 6**. For bacteria (**Figure 6a**), soil pH, available nitrogen (AN), available phosphorus (AP), available potassium (AK), and organic matter (OM) exhibited strong positive correlations (*ρ* ≥ 0.8, *p* < 0.01) with *Actinobacteriota* (Phylum), *Burkholderiales* (Order), *Burkholderiaceae* (Family), and *Burkholderia* (Genus). Conversely, these same soil parameters showed strong negative correlations (*ρ* ≤ -0.8, *p* < 0.01) with *Subgroup 2* (Order to Genus). Additionally, *Acidobacteriae* (Class) was negatively correlated with AK (-0.8 ≤ *ρ* ≤ -0.6, *p* < 0.05). Among fungi (**Figure 6b**), 12 specific microbial taxa (including *Mortierellomycota, etc.*) demonstrated positive correlations (*ρ* ≥ 0.6, *p* < 0.05) with soil properties, particularly showing strong positive associations (*ρ* ≥ 0.8, *p* < 0.01) with soil pH, AN, AP, AK, and OM. In contrast, *Fusarium solani* (Species) displayed a negative correlation (*ρ* ≤ -0.6, *p* < 0.05). Collectively, soil pH, OM, N, AN, AK, and AP emerged as key determinants governing the composition and abundance of some specific bacterial and fungal taxa in the soil ecosystem.

### 3.7 Correlations among the citrus yield and quality, soil properties, soil microorganism, and SGM intercropping years

**Figure 7:**
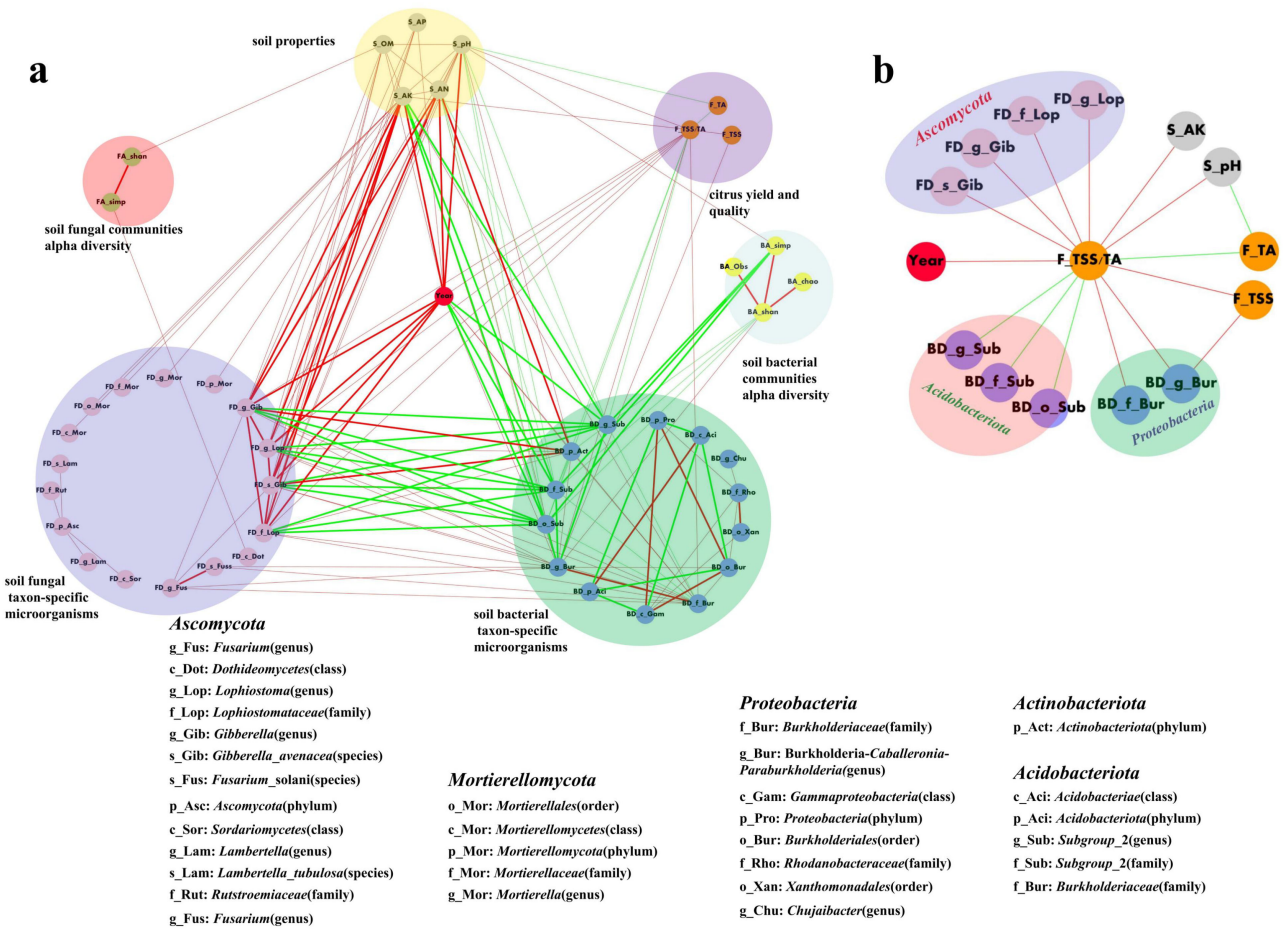
Correlation networks of **(a)** citrus yield/quality, soil properties, microbial α-diversity, and taxon-specific microbes (Spearman’s |*ρ*| ≥ 0.8, *p* < 0.01) and **(b)** key factors strongly correlated with citrus yield/quality (|*ρ*| ≥ 0.8, *p* < 0.01). Red/green lines indicate positive/negative correlations; bold lines represent strong correlations (|*ρ*| ≥ 0.9, *p* < 0.01).

The results of a network correlation analysis revealing multi-component interactions within the citrus orchard intercropped with SGM system are presented in **Figure 7** (Spearman’s |*ρ*| ≥ 0.8, *p* < 0.01). As illustrated in **Figure 7a**, intercropping duration (years) exhibited numerous direct and indirect correlations with soil properties, the taxon-specific microbes, soil microbial diversity, and fruit quality. Overall, extremely strong positive correlations (|*ρ*| ≥ 0.9, *p* < 0.01) were prevalent both among and within the core factors of intercropping duration (years), soil properties (specifically pH, available potassium, and available nitrogen), and some taxon-specific microbes. Notably, the taxon-specific microbes affiliated with *Acidobacteriota* and the titratable acidity (TA) index of fruit quality exhibited consistently negative correlations with all other analyzed factors, whereas all remaining correlations were positive. Additionally, soil properties and microbial factors exhibited several strong correlations (|*ρ*| ≥ 0.8, *p* < 0.01) with soil microbial diversity. Analysis of factors directly and strongly correlated with fruit quality (**Figure 7b**, |*ρ*| ≥ 0.8, *p* < 0.01) demonstrated that fruit quality was primarily influenced by three external factors: intercropping duration (years), soil properties, and some taxon-specific microbes. Notably, certain taxa within the phylum *Acidobacteria* exhibited negative correlations with fruit quality. Additionally, the titratable acidity (TA) index of fruits showed negative correlations with both soil pH and the TSS/TA. In contrast, all other pairwise relationships among these factors demonstrated positive correlations.

This study demonstrated that citrus fruit quality was predominantly influenced by intercropping duration (years), key soil properties (pH and available potassium, AK), and specific soil microbial taxa (*Ascomycota*, *Acidobacteriota*, and *Proteobacteria*), exhibiting highly significant strong correlations (|*ρ*| ≥ 0.8, *p* < 0.01).

## 4 Discussion

Cropping systems significantly influence orchard soil ecosystems by altering physical characteristics, chemical properties, and microbial communities—factors critically determining crop performance and quality (Wang T. et al., 2022; Li C. et al., 2023; Duan et al., 2024). This study specifically examined how SGM intercropping duration affects citrus yield/quality parameters and soil ecosystem dynamics (physicochemical properties and microbial structure), providing actionable insights for sustainable orchard management.

### 4.1 Effect of intercropping SGM on soil properties

Extensive research has documented the critical role of leguminous green manure incorporation in enhancing soil fertility and promoting nutrient transformation (Dong et al., 2021; Wang T. et al., 2022; Fu et al., 2023; Duan et al., 2024; Qin et al., 2024). The current investigation revealed that 1-year SGM intercropping significantly enhanced most soil physicochemical properties, with the exception of total N, P, and K content. Further improvements in soil pH and key nutrient indicators (AN, AK, AP, OM) were observed after 2 years of intercropping (T2) compared to the first year (T1) (**Table 2**). This finding aligns with established evidence demonstrating the nitrogen-fixing capacity of legume crops and their soil amelioration effects (Fu et al., 2023; Qin et al., 2024). The practice of intercropping SGM application effectively regulates soil nutrient conversion rates, leading to significant improvements in multiple soil parameters including pH, organic matter content, and AN, AK and AP, etc., ultimately enhancing crop yield and quality (Wang T. et al., 2022; Duan et al., 2024; Qin et al., 2024). The duration of SGM intercropping in orchards showed a positive correlation with the enhancement of essential nutrient conversion efficiency. These soil modifications, particularly the elevation of acidic soil pH and nutrient enrichment, subsequently influenced microbial community diversity and composition (Li et al., 2020; Fu et al., 2023; Qin et al., 2024). Consequently, SGM intercropping demonstrated substantial benefits for soil physicochemical properties in citrus orchards, with progressively more pronounced effects on soil pH neutralization and fertility improvement as intercropping duration (years) increased (**Table 2**).

### 4.2 Effect of intercropping SGM on soil microbial community

As the most biologically active and taxonomically diverse components of soil ecosystems, microorganisms perform indispensable ecological functions, including organic matter decomposition, biogeochemical cycling (Li et al., 2020; Adomako et al., 2022; Li C. et al., 2023), and modulation of plant growth and disease resistance (Shi et al., 2019; Philippot et al., 2024). Critical microbial community parameters—encompassing composition, α/β-diversity, phylogenetic structure, keystone taxon abundance, and co-occurrence network topology—serve as robust bioindicators for assessing soil ecosystem health and functional capacity (Li et al., 2020; Li C. et al., 2023; Li J. et al., 2023).

#### 4.2.1 Effect on soil microbial community composition and diversity

Numerous studies have demonstrated that green manure intercropping significantly alters microbial community composition, diversity, and structure (Dong et al., 2021; Wang T. et al., 2022; Fu et al., 2023; Duan et al., 2024; Qin et al., 2024). The results demonstrated that SGM intercropping in citrus orchards induced marked changes in soil microbial communities and associated physicochemical properties (**Figure 1**). Taxonomic analysis identified *Acidobacteria*, *Proteobacteria*, *Chloroflexi*, *Actinobacteriota*, and *Gemmatimonadota* as dominant bacterial phyla, while *Ascomycota*, *Mortierellomycota*, and *Basidiomycota* predominated among fungi—consistent with global soil microbial distributions (Wang T. et al., 2022; Duan et al., 2024; Philippot et al., 2024). Notably, SGM intercropping practices induced differential shifts in microbial composition (**Figure 1a**) characterized by (i) increased *Actinobacteria* and decreased *Acidobacteria* in bacterial communities and (ii) elevated *Mortierellomycota* and reduced *Ascomycota* abundance in fungal populations (**Figure 2**). The results demonstrated that bacterial communities exhibited concurrent enrichment of *Actinobacteria* and *Proteobacteria* phyla alongside the depletion of *Acidobacteriota*, while fungal communities showed concomitant increases in *Mortierellomycota* abundance with reductions in *Ascomycota* (**Figure 2**).

Many researches have established soil pH and nutrient availability as primary determinants of microbial community dynamics (Kyaschenko et al., 2017; Lin et al., 2019; Wang Z. et al., 2022; Philippot et al., 2024; Raiesi et al., 2024). Our findings corroborated these observations, demonstrating that available nitrogen (AN), available phosphorus (AP), available potassium (AK), organic matter (OM), and pH constituted the key edaphic factors governing microbial succession following soybean green manure (SGM) incorporation (**Figures 4**, **5**; **Supplementary Table S10**). The soil fertility index, which positively correlates with nutrient levels, emerged as a robust predictor of microbial diversity. Statistical analyses revealed significant positive correlations (*p* < 0.01) between these soil parameters (particularly pH, AN, AP, AK, and OM) and both bacterial and fungal community diversity metrics (**Figure 5**). Consistent with previous reports (Duan et al., 2024; Xiao et al., 2025), soil properties exhibited stronger regulatory effects on bacterial communities, with pH, AN, AP, AK, and OM all showing substantial correlations (*ρ* ≥0.6) with bacterial Shannon diversity. In contrast, fungal diversity demonstrated significant correlations (*ρ* ≥ 0.6) only with AN and OM concentrations (**Figure 5**).

In total, the observed changes in soil properties (particularly pH and fertility indices) showed significant associations with alterations in soil microbial community composition and diversity under SGM intercropping systems in citrus orchards.

#### 4.2.2 Effect on the co-occurrence network

The co-occurrence network analysis revealed that SGM intercropping in citrus orchards significantly enhanced the organizational complexity of soil microbial communities as evidenced by increased network nodes, connections, and species richness following SGM introduction (**Figure 3**; **Supplementary Tables S8**; **S9**). Research indicates that increases in node count, connectivity, and taxonomic richness within co-occurrence networks signify enhanced ecosystem health while simultaneously introducing novel ecological challenges. The core value of such network expansion lies in achieving equilibrium between functional augmentation and risk mitigation through structured development rather than pursuing quantitative complexity alone (Lei et al., 2023; Zhu et al., 2023; Qiao et al., 2024; Zhai et al., 2024).

A balance of positive and negative connections contributes to network stability (Luo et al., 2023). The study revealed a balanced distribution of positive and negative connections within the soil bacterial and fungal co-occurrence network following SGM intercropping, with this equilibrium being particularly pronounced under the 2-year intercropping regime (T2) (**Figure 3**; **Supplementary Table S7**). It is noteworthy that the negative connectivity number of the fungal co-occurrence network in the 1-year intercropped treatment was zero, which may be due to the *Fusarium* wilt disease of soybean in the 1-year intercropped SGM treatment group affecting the soil microflora fungal community environment (**Figures 2c, d**) (Zhou et al., 2022; Mendes et al., 2023). Despite the absence of documented *Fusarium* wilt cases in citrus to date, this potential pathogen requires increased attention. Furthermore, planting SGM in acid-biased environments tends to lead to the development of *Fusarium* wilt diseases in leguminous crops and has impacted the structure and network of soil fungal communities (Zhou et al., 2022; Du et al., 2024).

Small-world networks of interactions are characterized by short path lengths and have been shown to respond rapidly to disturbances in ecosystems (Zhou et al., 2010). This study showed that with increasing years of SGM intercropping, the average path length of bacterial co-occurrence networks decreased, while an opposite trend was observed in fungal communities (**Supplementary Table S7**). Therefore, intercropping soybean crops and using them as green manure led to an increase in the complexity of the bacterial and fungal communities in orchard soils, with enhanced internal interactions within the bacterial communities and weakened internal interactions within the fungal communities, which tended to be looser. These results are consistent with the results of Duan et al (Duan et al., 2024), who showed an increase in the interactions of bacterial networks and a strengthening of modularity of fungal networks and a loosening of internal interactions within the overall network after intercropping of a leguminous green manure in a tea garden.

#### 4.2.3 Effect on soil-taxon-specific microbes

The SGM intercropping system induced distinct shifts in microbial community composition (**Figure 1a**), demonstrating selective recruitment of specific taxonomic groups (**Figure 2**). Notably, SGM treatment recruited *Actinobacteria* and *Proteobacteria* while reducing *Acidobacteria* in bacterial communities. Fungal communities exhibited increased *Mortierellomycota* abundance with concomitant reduction in *Ascomycota* populations (**Figure 2**).

The correlation analysis (**Figure 6**) between soil properties and taxon-specific microbes (**Figure 2**) revealed that *Actinobacteria*, *Proteobacteria*, *Mortierellomycota*, and *Ascomycetes* (excluding *Fusarium solani*) showed significant positive correlations with soil properties including pH, OM, AN, AP, AK, and N (|*ρ*| ≥ 0.6, *p* < 0.05). In contrast, *Fusarium* and *Acidobacteria* exhibited opposite trends. Previous studies have demonstrated that *Acidobacteria* dominate in acidic soils, with their relative abundance increasing as pH decreases (Rousk et al., 2010; Zhao et al., 2025). These bacteria also play crucial roles in soil nutrient cycling and ecological function regulation. *Actinobacteria* and *Proteobacteria* contribute significantly to soil multifunctionality (Ma et al., 2022; Shi et al., 2025). *Proteobacteria* are particularly important for organic phosphorus activation (Chen et al., 2024) and community structure stabilization (Liu et al., 2025). *Actinobacteria* regulate microbial community structure, mediate nutrient transformation and plant uptake, and participate in organic pollutant degradation and heavy metal redox processes, making them essential for soil improvement, biomass maintenance, and pollutant remediation (Ma et al., 2022; Cui et al., 2023; Sun et al., 2024). *Ascomycetes* and other fungal groups serve as key indicators of soil health (Sun et al., 2024), while *Mortierellomycota* contribute to straw degradation and soil nutrient cycling through their ability to break down hemicellulose, cellulose, and lignin (Ning et al., 2022 2022). Notably, the 1-year intercropped SGM treatment group showed elevated recruitment of *Fusarium solani*, a known causal agent of soybean *Fusarium* wilt that often leads to significant crop losses (Ellis et al., 2016). Although no *Fusarium*-related disease symptoms were observed in citrus, soil acidification, barrenness, and nutrient imbalance have been identified as predisposing factors for soybean *Fusarium* wilt (Ning et al., 2020 2020; Osorio and Habte, 2014 2014). This may explain the establishment and dominance of *F. solani* in the T1 group, suggesting that *Fusarium* wilt in soybean crops intercropped with citrus warrants careful attention.

The SGM intercropping system demonstrated significant temporal effects on soil microbial community composition. Long-term SGM intercropping consistently enriched beneficial phyla including *Actinobacteria*, *Proteobacteria*, *Ascomycota*, and *Mortierellomycota* while reducing the relative abundance of *Acidobacteria*. This selective microbial recruitment pattern correlated with enhanced soil quality, greater microbial community functional versatility, and improved stability of the ecological network.

### 4.3 Effect of intercropping SGM on citrus quality

Intercropping with leguminous crops and green manure incorporation represent high-yielding, ecologically beneficial, and sustainable cultivation practices that enhance crop growth while improving the soil quality and microbial communities (Wang T. et al., 2022; Duan et al., 2024; Fan et al., 2025). Being consistent with this principle, the results of this study showed that SGM intercropping enhanced citrus fruit yield and quality parameters. Notably, after 2 years of implementation, this treatment significantly increased the total soluble solids (TSS) and the TSS/TA ratio in citrus fruits relative to the non-intercropped control (**Table 1**).

An analysis of factors exhibiting strong correlations with citrus fruit quality (Spearman coefficient |*ρ*| ≥ 0.8, *p* < 0.01; **Figure 7b**) revealed that intercropping duration with SGM significantly and positively correlated with the TSS/TA ratio—a key indicator of flavor maturity in citrus. This aligns with documented evidence that long-term green manure application enhances fruit quality (Zhang et al., 2022; Yang et al., 2023). Concurrently, soil nutrient availability demonstrated strong positive correlations with multiple fruit quality parameters, consistent with established mechanisms whereby green manure improves edaphic conditions to elevate crop quality (Wang T. et al., 2022; Zhang et al., 2022; Duan et al., 2024). Contrastingly, titratable acidity (TA) showed a significant negative correlation with soil pH (*p* < 0.01). Critically, titratable acidity (TA) serves as a negative indicator of fresh fruit palatability (China National Standardization Administration Committee, 2008; Li et al., 2014; Yang et al., 2014), further corroborating the interdependent relationship between soil properties and sensory quality attributes. Notably, specific microbial taxa—predominantly affiliated with *Actinobacteria*, *Proteobacteria*, *Acidobacteriota*, and *Ascomycota*—exhibited robust correlations with quality indices (**Figure 7**). Previous studies have shown that *Proteobacteria* and *Ascomycota* are positively associated with crop performance (Ning et al., 2022; Wang T. et al., 2022; Duan et al., 2024). Despite the positive role of *Acidobacteria* in participating in nutrient activation and organic matter degradation, as well as in enhancing primary productivity, many *Acidobacteria* have been associated with oligotrophic strategies and have successfully proliferated in low-nutrient environments, especially with poor soil quality (Kielak et al., 2016; Stone et al., 2023). Collectively, these findings underscore that SGM intercropping synchronizes soil physicochemical properties, microbiome dynamics, and fruit quality optimization, with intercropping duration being a pivotal regulatory factor.

Therefore, this study underscored that the duration of sustained intercropping with SGM significantly impacts soil physicochemical properties and microbial communities within citrus orchards and consequently reveals intricate links to the formation of key fruit quality attributes in citrus.

In general, in this study, intercropping SGM contributed to improved soil physicochemical properties (**Table 2**) and modified soil microbial community structure (**Figures 1**–**6**), consequently enhancing citrus fruit quality (**Table 1**; **Figure 7**). However, due to the short duration (2 years) of the SGM treatment, the observed improvements in citrus fruit yield and titratable acidity (a key quality parameter in **Table 1**) were not statistically significant. Consequently, extending SGM intercropping beyond the current trial duration may augment beneficial effects on orchard ecological functions (fruit yield and quality) and soil ecosystem attributes (soil properties and microbial community structure), rendering these positive trends more pronounced.

## 5 Conclusion

This study revealed that soybean green manure (SGM) intercropping in acidified citrus orchards effectively modulated soil characteristics, reshaped microbial communities, enhanced biodiversity, optimized network complexity, and intensified microbial interactions, resulting in superior fruit quality. After 2 years of SGM intercropping, significant improvements were observed in orchard soil pH and fertility, along with the selective recruitment of beneficial microorganisms (*Proteobacteria*, *Actinobacteria*, *Ascomycota*, and *Mortierellomycota*), while reducing acidophilic *Acidobacteria* populations. These changes led to increased complexity in both bacterial and fungal communities, strengthened microbial network interactions, and consequent enhancement of fruit quality parameters. Importantly, significant correlations were established between fruit quality improvement and specific changes in soil physicochemical parameters, microbial community structure, and the recruitment of some keystone microbial taxa. These findings provide fundamental insights into the ecological mechanisms underlying soil environment improvement in acidified citrus orchards through green manure intercropping, suggesting this approach as a promising strategy for sustainable citrus production in acidic regions. Thus, future research should explore the long-term effects of SGM intercropping on citrus rhizosphere microbiome dynamics and soil carbon sequestration while evaluating its economic feasibility across different acidic soil types and climatic conditions.

## Data Availability

The original contributions presented in the study are publicly available. This data can be found here: National Center for Biotechnology Information (NCBI) BioProject database under accession number: PRJNA957046.
