# Supplementary material for: Soybean green manure intercropping improves citrus quality by improving soil quality and altering microbial communities
Source: Front Plant Sci. 2025 Aug 28;16:1560550. doi: 10.3389/fpls.2025.1560550 (PMC12424052; doi:10.3389/fpls.2025.1560550)
Supplement: Supplementary file 1 [file Supplementaryfile1.zip › Supplementary File 1/supplementary figures.doc]

# Soybean green manure intercropping improves citrus quality by improving soil quality and altering microbial communities

Sufeng Deng et al.

Supplementary Figure 1 Bacterial community dilution curve (after drawing).

Supplementary Figure 2 Fungal community dilution curve (after drawing).

Supplementary Figure 3 The proportion of nodes in the co-occurrence network of soil bacterial communities at phylum taxon.

Supplementary Figure 4 The proportion of nodes in the co-occurrence network of soil fungal communities at phylum taxon.
